# Efficacy and safety of fluzoparib combined with anlotinib in extensive stage small cell lung cancer after first-line platinum-based chemotherapy: a multi-center, single-arm prospective phase II clinical study (STAMP study)

**DOI:** 10.1186/s12885-023-11230-5

**Published:** 2023-08-14

**Authors:** Deyu Li, Zhangzhou Huang, Jiangming Zhong, Li Lin, Guifeng Zhang, Wu Zhuang, Zhenhua Liu

**Affiliations:** 1 Department of Medical Oncology, Provincial Clinical College, Fujian Medical University, Fujian Provincial Hospital, NO.134 Dongjie Street, Fuzhou, 350001 Fujian China; 2 Department of Thoracic Oncology, College of Clinical Medicine for Oncology, Fujian Medical University, Fujian Cancer Hospital, NO.420 Fuma Road, Fuzhou, Fujian 350000 China

**Keywords:** Fluzoparib, Anlotinib, Small cell lung cancer, Protocol

## Abstract

**Background:**

Small-cell lung cancer (SCLC) is a highly aggressive and lethal malignancy that accounts for 10–15% of lung cancers, and it is generally divided into limited and extensive stage. The standard of care for patients with newly diagnosed extensive-stage SCLC (ES-SCLC) is still platinum-based chemotherapy and as maintenance therapy scheme. Although most parts of patients experience a significant tumor response to first-line therapy, the disease recurs invariably. Anlotinib hydrochloride, a novel oral multitarget tyrosine kinase inhibitor, has significant inhibitory activity against angiogenesis-related kinases, such as VEGFR, FGFR, PDGFR, and c-Kit kinase associated with tumor cell proliferation. Fluzoparib is a type of inhibitor of poly ADP ribose polymerase (PARP, including PARPl, PARP2 and PARP3). Previous studies have shown that Fluzoparib has a strong inhibitory effect on PARP1 activity at the molecular and cellular levels.

**Methods:**

This is a multi-center, prospective, single-arm phase II clinical study. A total of 50 ES-SCLC patients who experienced disease progression after first-line standard platinum-based chemotherapy with/without immune checkpoint inhibitors scheme, or within 6 months after the completion of treatment will be recruited. Those who had prior treatment with any PARP inhibitor or antiangiogenic agent includes anlotinib, bevacizumab, sorafenib, and thalidomide are excluded. Eligible patients will receive oral anlotinib 8 mg once daily and oral fluzoparib 150 mg twice daily until disease progression or intolerable toxicity. The primary endpoint is objective response rate (ORR).

**Discussion:**

The addition of fluzoparib to anlotinib is expected to increase the clinical benefit in ES-SCLC patients after platinum-based chemotherapy.

**Trial registration:**

This study protocol was prospectively registered on June 17, 2021. ClinicalTrials.gov Identifier: NCT04933175.

## Background

In recent years, people have made large efforts in improving the existing maintenance treatment strategies for ES-SCLC patients after the standard first-line chemotherapy. The poor prognosis and no preferable options may be largely related to chemotherapy drug resistance. Non-activating mutations of *TP53* and *RB1* are the most common mutations in SCLC, regrettably, no definite target is confirmed to be useful till now. Newly diagnosed SCLC experience a significant tumor response to first-line platinum-based chemotherapy with a response rate of 50–70%. Sensitivity to platinum-based chemotherapy may be predicted via determining interval from the end of first-line chemotherapy to disease progression, and it is considered to be correlated with second-line chemotherapy efficacy. Overall, the response rate of second-line therapy for SCLC is low at 5–30% [1, 2].

Recently, targeting DNA damage repair therapy including PARP inhibitors have shown therapeutic efficacy in SCLC and is considered to be a new potential treatment strategy for SCLC [3]. Although homologous recombination deficiency (HRD) or *BRCA1/2* gene mutations was not detected in SCLC very often, PARP inhibitors showed some antitumor activity in some preclinical and early clinical studies. However, the tumor suppressive activity of PARP inhibitor alone in SCLC is relatively low. PFS of UK STOMP study using olaparib as a maintenance therapy after first-line treatment for SCLC failed to be improved [4]. Therefore, PARP inhibitors in combination with other antitumor agents are emerging as a new clinical research strategy. One phase I/II clinical study published in Cancer Discovery in 2019 enrolled 50 SCLC patients given olaparib 200 mg bid × 7d combined with temozolomide 75 mg/m2 QD × 7d Q3W after the failure of previous standard therapy, and the result presented an ORR of 41.7%, a median PFS of 4.2 months and a median OS of 8.5 months [5]. Tumor response was confirmed in olaparib in combination with PD-L1 monoclonal antibody durvalumab in second-line and beyond in SCLC, especially two of three patients with inflammatory phenotype achieved PR [6]. Based on the results of ALTER1202 [7], the CFDA approved anlotinib for third-line and beyond treatment of SCLC, and lots of clinical studies of anlotinib in combination with other antitumor drugs for second-line and beyond treatment of SCLC are conducted, including anlotinib combined with chemotherapy (ChiCTR1500030474, ChiCTR1900028430, ChiCTR1900021983, NCT03823118), anlotinib combined with immunotherapy (NCT04192682, NCT04055792, NCT04234607).

Preclinical studies showed that tumor vascular regression and tissue hypoxia caused by anti-angiogenic therapy increase the sensitivity of tumor cells to PARP inhibitors, suggesting a synergistic anti-tumor effect of PARP inhibiting and anti-angiogenic therapy [8, 9]. In addition, olaparib combined with Bevacizumab for solid tumors also showed anti-tumor activity in phase I clinical study with no serious adverse events related to olaparib or dose-limiting toxicities [10]. A phase II randomized placebo-controlled study of olaparib in combination with the pan-inhibitors of VEGFR cediranib for the second-line treatment of ovarian cancer showed a significant prolongation of median PFS compared to olaparib alone (17.7 months versus 9.0 months, HR = 2.9, *P* = 0.001) [11], which is suggested that combined therapy of PARP inhibitors and pan-inhibitors of VEGFR may have synergistic antitumor effect.

Anlotinib is also a pan-inhibitor of VEGFR, which also inhibits *PDGFR*, *FGFR*, c-*KIT* and other targets, and would have synergistic antitumor effect in combination with the PARP inhibitor fluzoparib theoretically. However, till now no clinical study on the combination of anlotinib and fluzoparib have been reported or registered. Pharmacokinetically, anlotinib is not the substrates of P-glycoprotein, mainly metabolized via CYP1A2 and CYP3A4/5, and mostly excreted in urine and feces. In terms of drug interactions, anlotinib has a moderate inhibitory effect on CYP3A, CYP2B6, CYP2C8, CYP2C9 and CYP2C19. Grade 3 or higher adverse reactions are mainly hypertension, hand-foot skin reaction and proteinuria. Fluzoparib is a substrate of the efflux transporter P-glycoprotein mainly metabolized via CYP3A4, and is mostly excreted as metabolites in urine and feces. In terms of drug interactions, fluzoparib has a relatively small effect on the inhibition of each CYP enzyme subtype and does not induce CYP3A4 or CYP1A2. The most common grade ≥ 3 treatment-emergent adverse events reported in the fuzuloparib group were anemia (25.1%), decreased platelet count (16.8%), and decreased neutrophil count (12.6%) [12]. The above pharmacokinetic studies showed metabolic transformation is the main pathway of anlotinib elimination, while renal excretion via urine was the main excretion route and fecal excretion was the secondary excretion route of fuzuloparib, therefore, the toxicity of the two drugs should not add up theoretically. In addition, real-world study data for a small number of patients treated with fluzoparib in combination with anrotinib showed no significant increase in toxic and side effects.

The combination of anlotinib and fluzoparib may theoretically decrease the metabolism of fluzoparib, increase its concentration in the blood and area in the drug curve. Therefore, fluzoparib dose should be reduced appropriately. Combination of anlotinib and fluzoparib would not make adverse events and not increase the incidence of ≥ grade 3 adverse events. In theory, it is feasible in combination of the anlotinib and fluzoparib.

The study protocol and informed consent of participant were discussed and approved by the Fujian Provincial Hospital Ethics Committee to fully protect the rights of participants, and the aim of the protocol is to explore the efficacy and safety of fluzoparib combined with anlotinib in the second-line treatment of patients with recurrent/extensive stage SCLC.

## Methods/design

### Study design

**Fig. 1:**
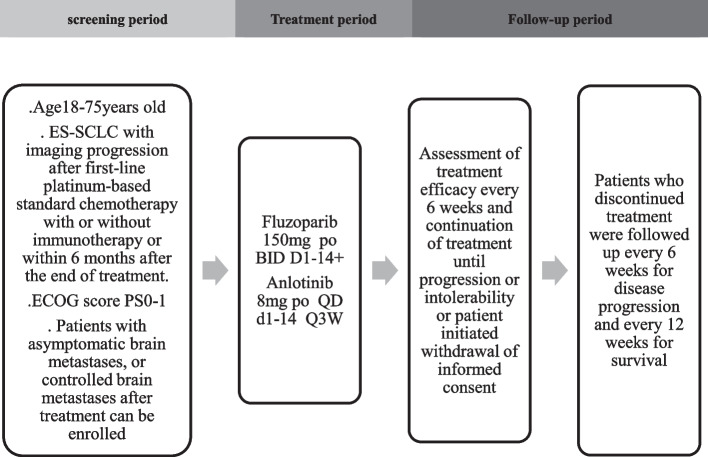
Study design

This is a multi-center, prospective, single-arm phase II clinical study (Fig. 1). A total of 50 ES-SCLC patients who have received standard platinum-based (two of cisplatin, carboplatin or lobaplatin) chemotherapy, and been considered to have disease progression assessed by imaging during the treatment period or within 6 months after the end of the last treatment according to RECIST1.1. will be recruited. The study is being conducted in accordance with the Declaratiion of Helsinki and Good Clinical Practice. The protocol and its amendments have been approved by local institutional review boards of all participating centers. The recruitment was started in May 2021.The enrolment is estimated to be completed in May 2022.

### Eligibility criteria

The patient inclusion and exclusion criteria are detailed in Table 1.

**Table 1:** Eligibility criteria

**Inclusion criteria**	Subjects who must meet all of the following inclusion criteria can be enrolled in this study
1	Be voluntarily enrolled in the study with signed informed consent, good compliance.
2	Age ≥ 18 years, age ≤ 75 years, regardless of gender.
3	Histologically and radiologically proved ES-SCLC according to the Veterans Adminstration Lung Study Group staging.
4	According to RECIST1.1 [13], at least one measurable lesion, and can be accurately measured at the baseline, the longest diameter of the baseline period ≥ 10 mm (in the case of lymph nodes, a short diameter ≥ 15 mm is required) without local treatment previously such as irradiation or tissue biopsy during the screening period. The selected measurement method is suitable for repeated measurement accurately, which can be computed tomography (CT) or magnetic resonance scan (MRI). If there is only one measurable suitable lesion, a baseline assessment of the tumor lesion should be performed at least 14 days after the diagnostic biopsy after acceptance as a target lesion.
5	Have received standard platinum-based (two of cisplatin, carboplatin or lobaplatin) chemotherapy, and been considered to have disease progression assessed by imaging during the treatment period or within 6 months after the end of the last treatment according to RECIST1.1.
6	The Eastern Cooperative Oncology Group (ECOG) physical status score of 0–2 with a minimum expected survival of 12 weeks.
7	Peripheral hemogram and liver and kidney function should achieve the following permissible limits (tested within 7 days prior to treatment initiation): White blood cells (WBC) ≥ 3.0 × 109/L or neutrophils (ANC) ≥ 1.5 × 109/L; (a) Hemoglobin (HGB) ≥ 80 g/L; (b) Platelets (PLT) ≥ 80 × 109/L; (c) Hepatic transaminases (AST, ALT) < 2.5 times the high limit of the normal range.; (d) Total bilirubin (TBIL) < 2 times the high limit of the normal range; (e) Creatinine (CREAT) < 1.5 times the high limit of the normal range.
8	Doppler ultrasound assessment: left ventricular ejection fraction (LVEF) ≥ low limit of normal (50%).
9	Women of childbearing age should take appropriate contraceptive measures from the beginning of screening to 3 months after stopping the treatment and should not breastfeed. No risk of pregnancy should be proved by a negative pregnancy test (urine or serum) within 7 days before the start of administration, or one of the following criteria a. Postmenopausal, defined as age ≥ 50, and amenorrhea for at least 12 months after cessation of all exogenous hormone replacement therapy. b. Postmenopausal age ≤ 50, and amenorrheic for 12 months or more after discontinuation of all exogenous hormone therapy, and luteinizing hormone (LH) and follicle-stimulating hormone (FSH) levels are within reference values for postmenopause. c. Previous irreversible sterilization, including hysterectomy, bilateral oophorectomy or bilateral salpingo-oophorectomy, not including bilateral tubal ligation.
10	Male patients should use barrier contraception (i.e., condoms) from beginning of screening to 3 months after stopping treatment.
11	Patients with asymptomatic brain metastases, or controlled brain metastases (clinical symptoms are stable for at least 2 weeks after symptomatic treatment for brain metastases). In addition, patients with prophylactic irradiation of the brain are allowed.
**Exclusion criteria**	Subjects are not eligible for enrollment in this study if they met any of the following criteria.
1	1. Have received any of the following treatments: a. Prior treatment with any PARP inhibitor or anti-angiogenic drug including anlotinib, avastin, sorafenib and thalidomide, etc. b. Have undergone major surgery within 4 weeks prior to the first dose. c. Have received more than 30% bone marrow irradiation or has received extensive radiotherapy within 4 weeks prior to the first dose.
2	Patients with uncontroled brainmets, or other malignant tumors, except basal cell carcinoma of the skin and carcinoma in situ.
3	The presence of significant cavitary lung tumors on imaging (CT or MRI).
4	Being treated in another clinical research study or having been treated in another clinical research study within 4 weeks prior to the start of the study.
5	Known hypersensitivity to the components of the study drug involved in the study.
6	Have received chemotherapy, radiotherapy or other experimental anticancer therapy (other than bisphosphonates) within 4 weeks prior to the first dose: those who have received prior local radiotherapy may be enrolled if the following conditions are met: the radiotherapy ended more than 4 weeks (more than 2 weeks for brain radiotherapy) from the start of study treatment; and the target lesion selected for this study is not in the radiotherapy area; or the target lesion is in the radiotherapy area but has been confirmed progression.
7	Patients who have not recovered from antineoplastic therapy-related adverse effects (except alopecia) to NCI-CTCAE ≤ grade 1 after prior systemic antineoplastic therapy.
8	Abnormal coagulation (INR > 1.5 or prothrombin time (PT) > ULN + 4 s or APTT > 1.5 ULN), with prone to bleeding or on thrombolytic or anticoagulant therapy; Note: The use of low-dose heparin for prophylactic purposes (adult daily dose of 0.6 to 1.2 10,000 U) or low-dose aspirin (dosage ≤ 100 mg daily) is accepted for prophylactic purposes.
9	Clinically significant hemoptysis (hemoptysis > ½ tablespoon per day) within 3 months prior to enrollment; or clinically significant hemorrhage symptom or prone to bleeding within 4 weeks prior to cohort, such as alimentary tract hemorrhage, hemorrhagic gastric ulcer (including gastrointestinal perforation and/or fistula, but the gastrointestinal perforation or fistula that has been surgically removed may be allowed), fecal occult blood (≥ + +) at baseline, unhealed wounds, ulcers, or fractures.
10	Renal insufficiency: urine protein ≥ + + , or 24-h urine protein amount > 1.0 g.
11	Patients with poor blood pressure control (systolic blood pressure ≥ 160 mmHg and diastolic blood pressure ≥ 100 mmHg) after drug therapy.
12	Patients with severe cardiovascular disease: grade II or higher myocardial ischemia or myocardial infarction, poorly controlled arrhythmias; grade III-IV cardiac insufficiency by NYHA criteria, or cardiac ultrasound suggestive of left ventricular ejection fraction (LVEF) < 50%.
13	The current presence of peripheral neuropathy ≥ CTCAE degree 2, except the cause of trauma.
14	Moderate or large uncontrolled plasma cavity effusions (including pleural, ascites, and pericardial effusions) via fluid aspiration, exacerbated chronic obstructive pulmonary disease, and respiratory disease in the active phase of pulmonary infection and/or acute bacterial or fungal infection that should accept intravenous antibiotic therapy.
15	There are factors that significantly affect absorption of oral drugs, such as inability to swallow, chronic diarrhea and intestinal obstruction.
16	Arterial/venous thrombotic events such as cerebrovascular accidents (including cerebral hemorrhage and cerebral infarction), deep vein thrombosis and pulmonary embolism that occurred within the 6 months prior to cohort.
17	Known history of psychotropic substance abuse, alcohol or drug abuse.
18	Active hepatitis (hepatitis B: HBsAg positive and HBV DNA ≥ 1 × 104 copies/ml; hepatitis C: HCV RNA positive and abnormal liver function) that cannot be controlled, or the combined infection of hepatitis B and C.
19	The positive blood or urine pregnancy result of women within 3 days prior to the first dose.
20	Poor compliance in the procedures and requirements of the study.
21	Any condition that will endanger patient's life or interference assessment results.
**Withdrawal from the study criteria**	
1	Withdrawal of informed consent form at any time by the subjects
2	Disease progressed assessed by medical imaging or clinical feature.
3	A pregnancy event occurs in the subject during the study
4	Other reasons that the trial treatment cannot be continued by the investigator The subject have to discontinue drug upon the occurrence of any of the following (including but not limited to)
**Criteria for termination of the study**	
1	An unintended, significant or unacceptable risk to the subject is identified
2	A significant failure of the protocol is discovered during trial execution
3	The investigational drug/trial treatment is ineffective, or it is not meaningful to continue the trial
4	Extreme difficulty in completing the trial due to serious delays in subject enrollment or frequent protocol deviations

### Intervention

Eligible patients will receive oral fluzopalib150mg twice daily and oral anlotinib 8 mg once daily until disease progression or development of intolerable toxic side effects. The dose giving and dose level adjustment are shown in Table 2.

**Table 2:** Dose adjustment criteria

NCI CTCAE 5.0		Management (after active treatment and observation)	Dose adjustment after the study drug is continued
**Anlotinib**			
**Abnormal liver function**	**Grade 1**	**Conduct follow-up as planned**	**8 mg, qd**
	**Grade 2 (normal baseline)**	**Delay dose, strengthen guarantees the liver treatment and close monitoring of liver function once a week till < grade 2 within 2 weeks, and then continue anlotinib at a lower dose level**	**8 mg, qod**
	**Grade 2 (abnormal baseline)**	**Strengthen guarantees the liver treatment and close monitoring of liver function once a week, continue anlotinib at an o initial dose**	**8 mg, qd**
	**Grade 3**	**Delay dose, strengthen guarantees the liver treatment and close monitoring of liver function twice a week till < grade 2 within 2 weeks, till < grade 2 within 2 weeks, and then continue anlotinib at a lower dose level**	**8 mg, qod**
	**Grade 4**	**Strengthen guarantees the liver treatment and close monitoring of liver function once/twice a week till < grade 2. Or have reasons for this issue**	**Discontinue anlotinib**
**Hypertension**	**SBP 120–139 mmHg or DBP 80–89 mmHg**	**Closely monitor, continue anlotinib at an initial dose**	**8 mg, qd**
	**Asymptomatic grade 2 hypertension**	**Continue anlotinib at an initial dose. Start taking antihypertension medication or adjust its dose till blood pressure is effectively controlled (SBP < 140 mmHg or DBP < 90 mmHg) within two weeks, otherwise dose can be reduced as per investigator’s discretion**	**8 mg, qd, or dose can be reduced as per investigator’s discretion**
	**Symptomatic grade 2 hypertension, or grade 3 hypertension**	**Discontinue anlotinib, and then start taking antihypertension medication or adjust its dose till blood pressure is effectively controlled within two weeks, continued anlotinib at an initial dose or a lower dose level**	**8 mg, qd, or 8 mg, qod as per investigator’s discretion**
**Abnormalities of bleeding and coagulation**	**Grade 1**	**Closely monitor, continue anlotinib at an initial dose**	**8 mg, qd**
	**Grade 2**	**Discontinue anlotinib, till AE ≤ 1, and then continue anlotinib at a lower dose**	**8 mg, qod**
	** ≥ Grade 3, or ≥ Grade 2 occurred twice (after discontinue or dose adjustment)**		**Withdraw from the pilot study**
**Fluozopalib**			
**Grade 3 hematological toxicity**	**Grade 3 leukopenia not accompanied by fever**	**Discontinue fluozopalib till Grade ≤ 2 hematological toxicity, then fluozopalib was continued at an initial dose level**	**150 mg twice daily**
		**While grade 3 hematologic toxicity occurred again, discontinue fluozopalib till grade ≤ 2 hematological toxicity, then fluozopalib was continued at a lower dose level**	**100 mg twice daily**
		**While grade 3 hematologic toxicity occurred third time, discontinue fluozopalib till grade ≤ 2 hematological toxicity, then fluozopalib was continued at a much lower dose level**	**50 mg twice daily**
	**Grade 3 neutropenia not accompanied by fever**	**Discontinue fluozopalib till Grade ≤ 2 hematological toxicity, then fluozopalib was continued at an initial dose level**	**150 mg twice daily**
		**While grade 3 hematologic toxicity occurred again, discontinue fluozopalib till grade ≤ 2 hematological toxicity, then fluozopalib was continued at a lower dose level**	**100 mg twice daily**
		**While grade 3 hematologic toxicity occurred third time, discontinue fluozopalib till grade ≤ 2 hematological toxicity, then fluozopalib was continued at a much lower dose level**	**50 mg twice daily**
	**Grade 3 neutropenia accompanied by fever**	**Discontinue fluozopalib till Grade ≤ 2 hematological toxicity, then fluozopalib was continued at a lower dose level**	**100 mg twice daily**
		**While occurred again, discontinue fluozopalib till grade ≤ 2 hematological toxicity, then fluozopalib was continued at a much lower dose level**	**50 mg twice daily**
	**Grade 3 leukopenia or neutropenia accompanied by grade 2 thrombocytopenia/grade 2 anemia**	**Discontinue fluozopalib till Grade ≤ 2 hematological toxicity, then fluozopalib was continued at a lower dose level**	**100 mg twice daily**
		**While occurred again, discontinue fluozopalib till grade ≤ 2 hematological toxicity, then fluozopalib was continued at a much lower dose level**	**50 mg twice daily**
**Grade 4 hematological toxicity**		**Discontinue fluozopalib till Grade ≤ 2 hematological toxicity, then fluozopalib was continued at a lower dose level**	**100 mg twice daily**
		**While occurred again, discontinue fluozopalib till grade ≤ 2 hematological toxicity, then fluozopalib was continued at a much lower dose level**	**50 mg twice daily**
**Grade 3 non-hematologic toxicity**		**Discontinue fluozopalib till Grade ≤ 1 hematological toxicity, then fluozopalib was continued at an initial dose level or a lower dose level**	**150 mg twice daily or 100 mg twice daily**
		**While occurred again, discontinue fluozopalib till grade ≤ 1 hematological toxicity, then fluozopalib was continued at a lower dose level**	**100 mg twice daily or 50 mg twice daily**
**Grade 4 non-hematologic toxicity**		**Discontinue fluozopalib till Grade ≤ 1 hematological toxicity, then fluozopalib was continued at a lower dose level**	**100 mg twice daily**
		**While occurred again, discontinue fluozopalib till grade ≤ 1 hematological toxicity, then fluozopalib was continued at a much lower dose level or discontinue**	**50 mg twice daily or discontinue**

*NCI CTCAE* National Cancer Institute Common Terminology Criteria for Adverse Events, *SBP* Systolic blood pressure, *DBP* Diastolic blood pressure

Take 1 capsule (8 mg) of anlotinib once daily on an empty stomach before breakfast. Stop 1 week after 2 consecutive weeks of oral administration, i.e. 3 weeks (21 days) as a treatment cycle. In case of missed dose, no additional dose will be given if the time for the next dose is confirmed to be shorter than 12 h. Patients with disease control (CR + PR + SD) and tolerable adverse effects will continue the dose.

For oral fluzoparib, it should be swallowed whole either after a meal or on an empty stomach (recommended after a meal). Take 3 capsules (150 mg) twice daily for 14 days, and 7 days of discontinuation as a treatment cycle (21 days). In case of a missed dose, the next dose should be taken normally at the scheduled time.

### Endpoints

The primary endpoint is Objective Response Rate. The secondary endpoints are progression-free survival, disease control rate, safety, duration of response, overall survival, quality of life. The safety endpoints are occurrence of adverse events, proportion of patients withdrawn due to adverse events, proportion of patients with dose adjustment due to adverse events. We will closely evaluate and monitor safety information in accordance with safety information evaluation and management practices during drug clinical trials. We will timely submit suspected and unexpected serious adverse reactions (SUSAR) reports through the pharmacovigilatory Electronic Transmission System (PV system), and timely submit safety update reports (DSUR) during the study and other potential serious safety risk information reports through the Drug Approval Center website. We will carry out risk monitoring, identification, assessment and control of safety information, timely discover existing safety problems or other risks, and promptly take measures of risk control and risk minimization, including general risk management measures (such as modifying clinical trial protocol), active suspension or termination of clinical trials.

Efficacy evaluation will be performed every two cycles (i.e., every 6 weeks) from 24 weeks after the first dose. All imaging data will be kept for patients with CR, PR, SD, and PD. If subjects discontinue the trial before the development of PD, follow up every 6 weeks for imaging (external hospital reports are acceptable) to observe whether the tumor progresses or take other anti-tumor therapies. Telephone or clinical follow-ups are acceptable.

### Statistical analysis

The sample size consideration was based on the unilateral exact test with α = 0.05 (unilateral), β = 0.20, and the second-line standard treatment plan recommended by the current guidelines for topotecan single-agent treatment of refractory SCLC patients with ORR of 9.4% [4]. There is 80% power to detect a significant difference of 9.4% in ORR (from 30%) between treatments based on a one-sided 5% significance level, 22 evaluable subjects need to be enrolled at least. Considering a dropout rate of 10%, a total of 25 subjects are expected to be enrolled. All things considered, 50 subjects are planned to be enrolled in this study. Median PFS and OS were estimated using Kaplan–Meier method. The two-sided statistical test is performed for statistical analysis.

Means, standard deviations, medians, maxima, and minima are listed for measurement data, and frequencies (composition ratio), rates, and confidence intervals are listed for count data and rank data. Statistical analyses were carried out with statistical software package SPSS 24.0. *P* less than 0.05 was considered as statistically significant. No data imputation will be performed for the missing data.

The mean, standard deviation, median, maximum and minimum values of quantitative data, such as age, height and weight are calculated for basic patient characteristics. The frequencies and percentages are listed for qualitative data, such as gender and ECOG scores.

## Discussion

This study will provide evidence on efficacy and safety fluzoparib combined with anlotinib in ES-SCLC, which may be used as a candidate standard therapy.

A previous study demonstrated anlotinib has a high efficacy and safety profile in patients with advanced NSCLC [14]. A preliminary phase III efficacy and safety study enrolled 439 patients were randomized 2:1 to the treatment group and the placebo group, and the treatment group received oral anlotinib 12 mg/d. Respectively, the PFS of the treatment group and the placebo group were 5.37 months and 1.4 months, the OS was 9.63 months and 6.3 months, and the ORR and disease control rate in the anlotinib group were 9.18% and 80.95% significantly better than those in the placebo group (0.7% and 37.06%). Adverse events in the anlotinib group including hypertension, proteinuria and hand-foot syndrome were mild or moderate and controllable. As the first effective vascular targeting drug for the monotherapy of advanced NSCLC, anlotinib targets include VEGFR1/2/3, FGFR1/2/3 and PDGFRα/β. Compared with Bevacizumab, anlotinib has a more comprehensive neovascular targets to achieve efficient inhibition of neovascularization.

A multicenter, randomized, double-blind study ALTER1202 enrolled 11 study centers nationwide was designed to observe 120 patients with pathologically confirmed SCLC who had been treated with at least two prior systemic chemotherapy regimens. The main efficacy indicator is PFS [15].At WCLC 2018, it announced that the study met the primary study endpoint with the PFS of 4.1 m vs 0.7 m [HR:0.19 (95% Cl:0.12–0.32), *p* < 0.0001] and ORR of 4.94% vs 2.63%, respectively, in the anlotinib group (82 patients) and the placebo group (38 patients) for survival benefit. At ESMO 2019, the final OS data for ALTER1202 continued to be presented that 78% of patients had OS events and OS data were mature with a median OS of 7.3 m (6.1–10.3) vs 4.9 m (2.7–6.0) [HR: 0.53 (95% Cl:0.34- 0.81), *p* = 0.0029], 6-month OS rate of 63.9% vs 32.7%, and 1-year OS rate of 30.6% vs 13.1%. Subsequent subgroup analysis of the 68 patients showed that patients who had received prior radiotherapy achieved significantly prolonged median PFS by up to 4.8 months in anlotinib group compared to placebo group (5.49 months vs. 0.69 months, HR 0.14, *P* < 0.0001), and also significantly prolonged OS (9.49 months vs. 4.89 months, HR 0.46, P = 0.0388).

Fluzoparib is an inhibitor of poly ADP ribose polymerase (PARP, including PARPl, PARP2 and PARP3). Previous studies showed that fluzoparib has a strong inhibitory effect on PARP1 activity at the molecular and cellular levels [16], and presents almost same activity as olaparib with IC50s of 2.0 nM and 1.5 nM at the molecular level and 8.0 nM and 4.9 nM at the cellular level (MDA-MB-436), respectively. PARP inhibitors could induce DNA damage, then increase the spontaneous formation of aggregation sites for Rad51 in homologous recombinant cells with normal function. In contrast, in BRCA1/2-deficient cells, with dysfunctional homologous recombination repair failed to induce the formation of aggregation sites for Rad51. The formation status of aggregation sites for Rad51 was examined by immunofluorescence while treated with fluzoparib and olaparib. The formation of aggregation sites for Rad51 was induced in normal BRCA1/2 cells (V-C8 #13–5) after treated with 30 μM of fluzoparib and olaparib in 24 h, whereas in defective BRCA1/2 cells (V-C8, MDA-MB-436, Capan-1 and SW-620), aggregation sites for Rad51 failed to be induced. The phosphorylation level of Anti-Phosphorylated Histone H2AX was detected to access the ability of DNA damage repair in BRCA1 mutant MDA-MB-436 cells when treated with fluzoparib in combination with temozolomide (TMZ). The phosphorylation of γ-H2AX significantly enhanced while in combination with fluzoparib and olaparib (the positive control compound) plus TMZ, and the result showed a significantly dose-dependence. These results revealed that fluzoparib and olaparib could inhibit DNA damage repair and synergize potentiating the DNA damage repair of alkylating agents such as TMZ with almost same activity.

In vitro studies showed that fluzoparib significantly inhibited the proliferation of V-C8 cell and MBA-MB-436 cell with an IC50 of approximately 100 nM and 1 μM, respectively. Fluzoparib and olaparib presented almost the same activity in V-C8 and MBA-MB-436 cells. In addition, fluzoparib could also intensify the inhibitory effect on cell proliferation via synergizing with TMZ, MMC or CDDP, and fluzoparib and TMZ showed the greatest synergy, which is about fourfold over EMZ alone [16].

The effect of fluozoparib on xenograft-tumors in nude mice with injected MX-1 cell (breast cancer BRCA1/2 mutation cell), MDA-MB-436 cell (breast cancer BRCA2 mutation cell)), capan-1 cell (pancreatic cancer cell with BRCA1 mutation) and SW-620 cell (colon cancer cell with reduced BRCA expression) was conducted [16], and the result showed significantly synergetic effect of fluzoparib and TMZ against the tumor cells above with obvious dose-dependent, and the synergetic effect may cause partial tumor regression. Synergetic effect of fluzoparib and TMZ would not increase toxic side effects except in the MX-1 tumor model (in which fluzoparib increased toxicity of TMZ, but recovered after drug discontinuance). Overall, the synergetic effect of fluzoparib on TMZ is almost the same as olaparib.

In clinical practice, parts of patients who are terrified of the chemotherapy due to the tolerability concerns, will be enrolled in the present study. What should be noted is that previously untreated ES-SCLC with failure of first-line platinum-based chemotherapy will receive fluzoparib plus anlotinib. Thus, this study will provide preliminary evidence of this combination therapy in the first-line setting. We wish that the present study will find a potent chemo-free treatment approach and bring new light of hope for ES-SCLC patients after failure of first-line platinum-based chemotherapy.

## Data Availability

Not applicable.

## Abbreviations

AE: Adverse events
CYP: Cytochrome P450 proteins
CR: Complete response
CT: Computed Tomography
CTCAE: Common terminology criteria for adverse events
DCR: Disease control rate
ECOG: Eastern Cooperative Oncology Group
DepOR: Depth of response
DOR: Duration of response
PARP: Poly ADP-ribose polymerase
VEGFR: Vascular endothelial growth factor receptor
LVEF: Left Ventricular Ejection Fraction
SCLC: Small cell lung cancer
ORR: Objective response rate
OS: Overall survival
OR: Odds ratio
PFS: Progression-free survival
BRCA1/2: Breast cancer susceptibility gene 1/2
ULN: Upper limit of normal
